# Case Report: Novel mutation in CHD4 triggers occult breast cancer with bone metastases

**DOI:** 10.3389/fonc.2025.1682794

**Published:** 2025-09-29

**Authors:** Hang Dong, Jingtong Zhao, Boyin Zhang, Xiaowen Wu, Guangyao Liu

**Affiliations:** ^1^ Department of Traumatic Orthopedics, Jilin University China-Japan Union Hospital, Changchun, China; ^2^ Department of Breast Surgery, Jilin University China-Japan Union Hospital, Changchun, China; ^3^ Department of Spine Surgery, Jilin University China-Japan Union Hospital, Changchun, Changchun, China; ^4^ Department of Genitourinary Oncology, Key Laboratory of Carcinogenesis and Translational Research (Ministry of Education/Beijing), Peking University Cancer, Beijing, China

**Keywords:** CHD4 mutation, occult breast cancer, bone metastases, NuRD complex, epigenetic modification

## Abstract

Chromatin domain-binding protein 4 (CHD4), the ATPase core component of the NuRD complex, exerts dual roles in epigenetic regulation—mediating both gene silencing and activation. We report a case of occult breast cancer with extensive bone metastasis harboring a novel somatic truncation mutation in the catalytic SNF2 domain (CHD4 p.Trp736Ter). This mutation, unreported in other cancers to date, abolishes ATPase activity and disrupts NuRD complex assembly. Multimodal analysis (PET/CT, biomarkers, pathology) confirmed the case as luminal-A subtype and revealed a significant response to endocrine therapy. We hypothesize that this CHD4 mutation may alter the protein’s dual regulatory balance, particularly enhancing its potential gene-activating functions, and propose that impaired chromatin remodeling driven by such mutations is associated with metastatic progression of breast cancer.

## 1 Introduction

CHD4, a dominant ATP-dependent chromatin remodeler within the NuRD complex, utilizes ATP hydrolysis to restructure nucleosomes (1, 2). As a core NuRD component, it recruits DNMTs to induce DNA methylation and partners with HDAC1/2, EZH2, and G9a to deposit repressive histone marks (H3K27me3/H3K9me2), silencing gene expression (3). Further evidence has emerged that the N-terminal region of CHD4 has gene-activating functions. These functions involve direct interaction with the ATPase domain of BRG1, which enhances transcriptional activity. This highlights the multifaceted role of CHD4 in epigenetic regulation (4). Beyond gene regulation, CHD4 is also essential for DNA damage repair and epigenetic reprogramming. Dysregulation profoundly impacts gene transcription and cellular processes.

CHD4 function relies on coordinated domain interactions. Pathogenic mutations disrupting these domains impair its activity, leading to defective DNA repair, aberrant gene expression, and tumorigenesis (5–7). Supporting this, a novel SNF2 domain nonsense mutation was identified in a patient with metastatic breast cancer (primary unknown, diagnosed via metastatic biopsies) who remains stable on endocrine therapy. Computational analysis suggests that this mutation has the potential to disrupt CHD4’s DNA-binding ability, which could in turn impair its gene-silencing function. Additionally, the mutation may also enhance its capacity for transcriptional activation. The integration of this case with existing research endeavors to elucidate the manner in which CHD4 mutations perturb its dual regulatory role and drive oncogenesis.

## 2 Clinical case

A 66-year-old postmenopausal woman with an unremarkable medical history presented with a diagnostically challenging metastatic tumor. Her clinical timeline began 35 years prior with a left acute mastitis episode requiring surgical incision and drainage, which evolved into chronic mastitis. In May 2022, she underwent ultrasound-guided closed drainage for a liver abscess, achieving full resolution. Seven months later in December 2022, acute-onset chest and back pain prompted abdominal CT imaging to evaluate potential liver abscess recurrence. While hepatic architecture appeared normal, the scan incidentally detected a T10 vertebral compression fracture. Subsequent spinal MRI revealed multiple osteolytic-osteoblastic mixed vertebral metastases, predominantly involving pedicles and posterior elements with characteristic rounded morphology. Serum tumor marker profiling was conducted to identify a primary malignancy. Carcinoembryonic antigen levels were notably elevated above reference ranges, whereas other markers including CA 15-3, CA 125, and alpha-fetoprotein remained within normal parameters. Despite extensive diagnostic investigations encompassing biochemical analyses and advanced imaging modalities, the origin of the primary tumor remains undetermined at this stage of evaluation.

### 2.1 Baseline 18F-FDG PET/CT imaging

**Figure 1:**
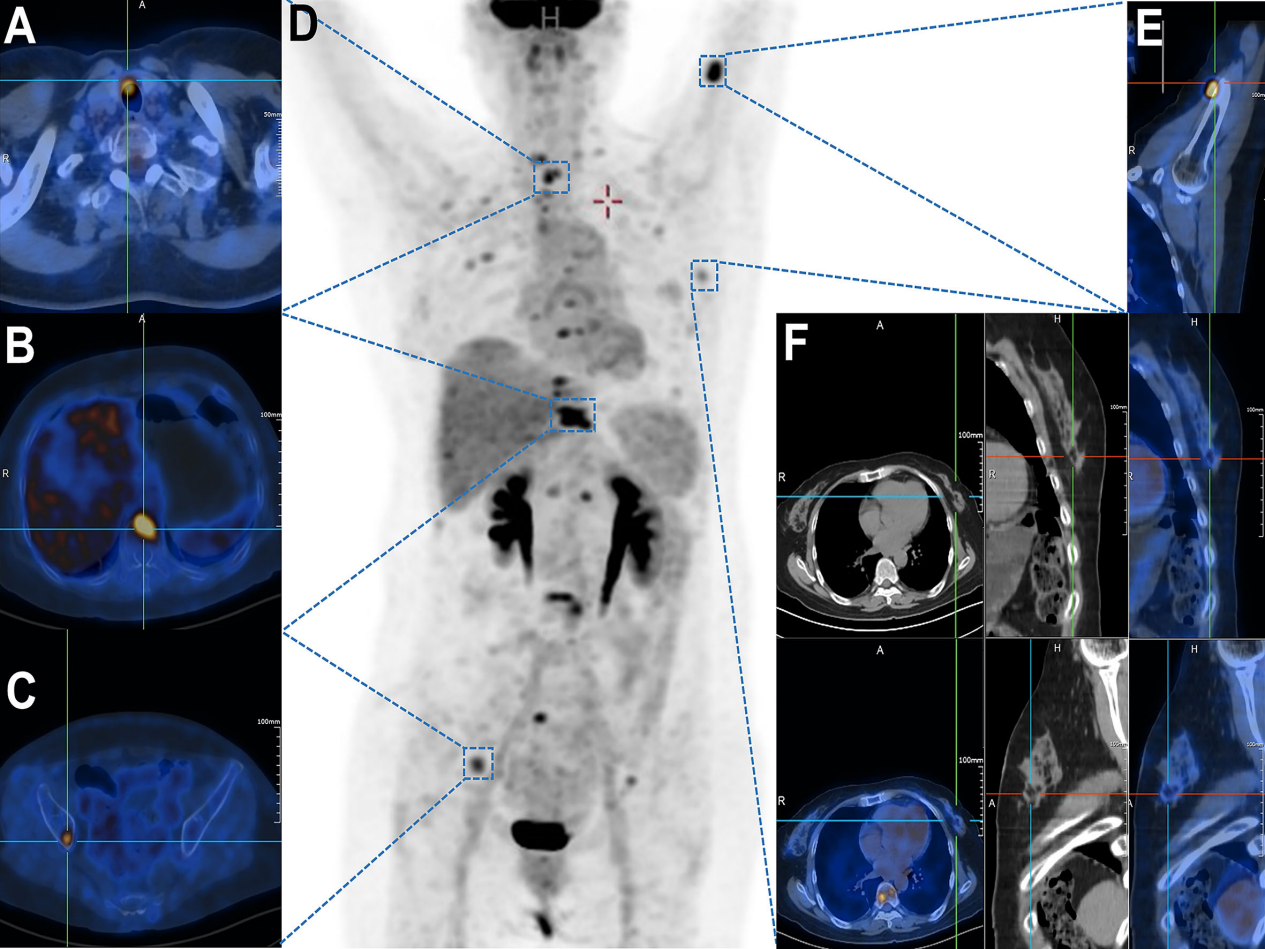
Whole-body ^18^F-FDG PET/CT demonstrating multifocal hypermetabolic lesions. **(A)** Thyroid isthmus lesion with focally increased FDG uptake (SUVmax = 9.41). **(B)** Vertebral metastasis at T10 level (SUVmax = 11.64). **(C)** Right iliac bone lesion showing elevated metabolic activity (SUVmax = [*value*]). **(D)** Maximum intensity projection (MIP) image revealing systemic dissemination of metabolically active metastases. **(E)** Left proximal humeral lesion with abnormal FDG avidity (SUVmax = [*value*]). **(F)** A suspicious mass in the left breast upper-outer quadrant demonstrated focal FDG avidity (SUVmax = 2.19) on axial, coronal, and sagittal fused PET/CT views.

18F-FDG PET/CT (**Figure 1D**) demonstrated a highly metabolic right thyroid nodule (13.0 × 6.7mm, SUVmax 9.41; **Figure 1A**) alongside FDG-avid lymphadenopathy in submandibular/cervical/supraclavicular (SUVmax 2.58) and mediastinal regions (SUVmax 4.19). Extensive hypermetabolic bone metastases were evident, including a pathological T10 fracture (SUVmax 11.64, CT HU 876; **Figure 1B**), right iliac lesion (SUVmax 6.32; **Figure 1C**), and left proximal humeral lesion (SUVmax 11.47; **Figure 1E**). A left breast nodule showed only borderline FDG uptake (SUVmax 2.19; **Figure 1F**), subsequently confirmed as benign (BI-RADS II) with periareolar fibrosis on ultrasound, consistent with chronic mastitis.

Breast cancer bone metastasis imaging: 18F-sodium fluoride PET/CT excels for osteoblastic metastases (alters management in 24–60% cases), while 18F-FDG PET/CT targets osteolytic disease. PET/CT is critical for advanced/high-risk staging, metastasis detection, NAC response, and recurrence surveillance (8). In suspected recurrence, PET/CT outperformed CECT/BS with perfect NPV, near-perfect AUC (0.99), and superior specificity (98.9%) for distant metastases, establishing it as a definitive standalone modality (9).

### 2.2 Longitudinal imaging progression of spinal metastases

**Figure 2:**
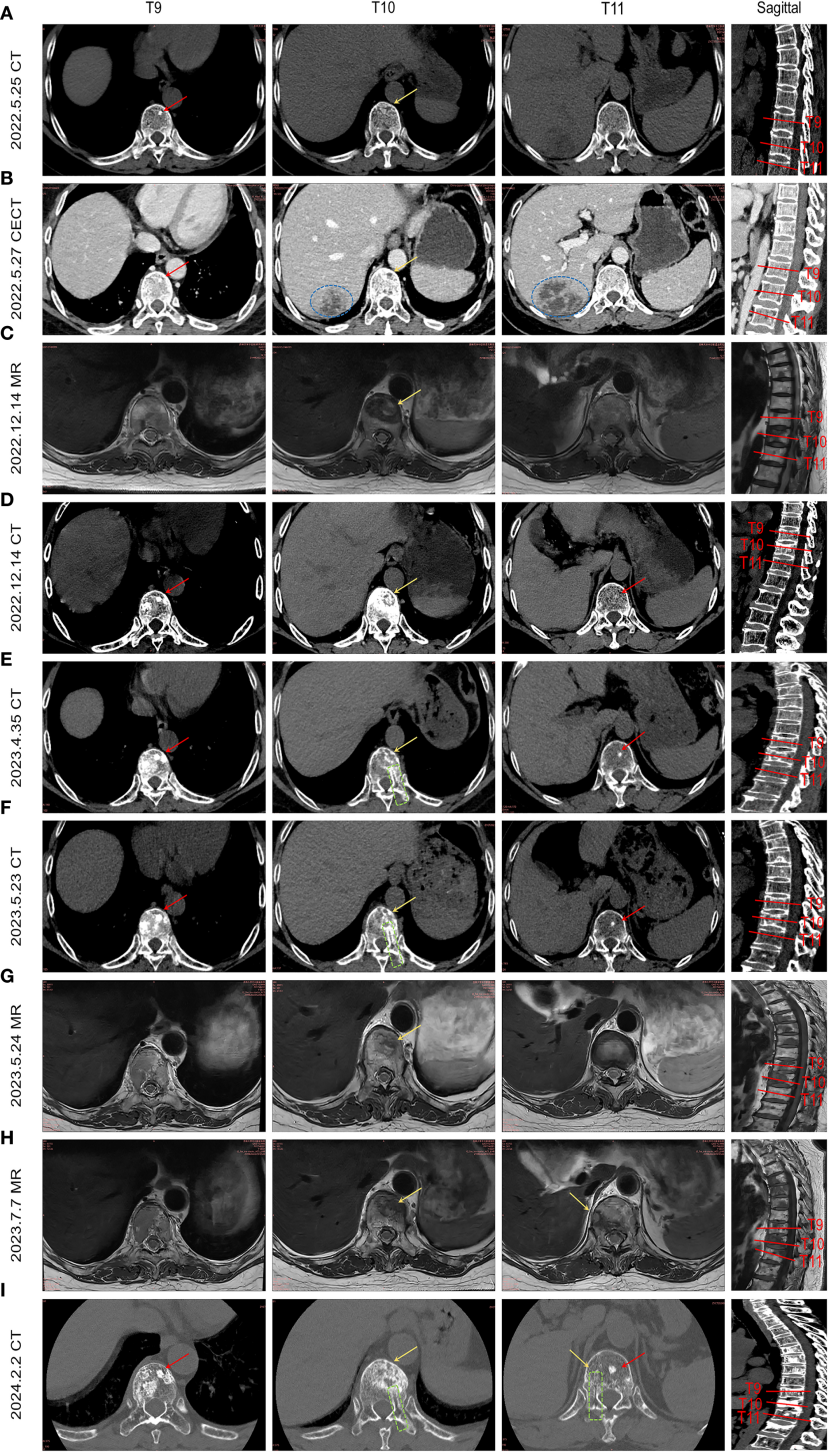
illustrates the temporal evolution of spinal metastasis in the patient. By integrating abdominal CT scans obtained during hospitalization for liver abscess and collating prior MRI/CT images, the imaging progression of breast cancer spinal metastases from onset to final outcome is presented sequentially. **(A)** Abdominal CT during liver abscess hospitalization shows a high-density shadow in the T9 vertebral body (red arrow) and worm-eaten changes at the anterior margin of T10 (yellow arrow). **(B)** Contrast-enhanced abdominal CT highlights the liver abscess (blue circle), T9 high-density shadow, and more pronounced worm-eaten changes at T10 anterior margin (yellow arrow). **(C)** Incidental MRI for low back pain reveals a metastatic lesion at T10 anterior margin (yellow arrow) and mixed signal intensity changes in T9. **(D)** Thoracic CT after 8 months shows multiple osteoblastic changes in T9 (red arrow), resorptive changes at T10 anterior margin, osteoblastic rim around osteolytic lesions, and new high-density shadows in T11. **(E, F)** Lesions progress with expanding osteoblastic foci in T9 and T11 (red arrows), enlarged worm-eaten changes at T10 anterior margin (yellow arrow), and a visible biopsy tract (green box). **(G, H)** Sagittal views show expanded T10 anterior lesions with compression fracture, metastatic involvement at T11 edge. **(I)** Eight-month follow-up thoracic CT after endocrine therapy demonstrates stabilized T9/T11 lesions (red arrows), improved T11 anterior worm-eaten changes (yellow arrow), no new compression fractures, and persistent biopsy tract (green box).

Serial imaging during the patient’s liver abscess hospitalization (initial abdominal CT: **Figure 2A**; follow-up CECT: **Figure 2B**) revealed incidental T9 vertebral body sclerosis (Red Arrow) and subtle T10 anterior margin moth-eaten destruction (Yellow Arrow), with T11 appearing unremarkable. These early metastatic features were only recognized retrospectively. Subsequent MRI during back pain episodes confirmed progressive T10 destruction (**Figure 2C**). Eight months post-metastasis, thoracic CT (**Figure 2D**) demonstrated T9 osteoblastic progression, T10 osteolysis with peripheral sclerosis, and new T11 sclerotic lesions. Follow-up CT (**Figures 2E, F**) showed expanding osteoblastic lesions at T9/T11 (Red Arrows), advancing T10 osteolysis (Yellow Arrows), and a visible biopsy tract (Green box). Concurrent MRI (**Figures 2G, H**) revealed T10 vertebral collapse and T11 involvement. Post-endocrine therapy imaging (**Figure 2I**) documented stabilized T9/T11 lesions, regressing T10 osteolysis, no new fractures, and persistent biopsy changes, demonstrating therapeutic response. This progression highlights breast cancer metastases’ heterogeneous radiographic presentation (osteolytic/osteoblastic/mixed), their subtle early imaging profile, and distinct growth patterns: osteoblastic foci exhibiting rapid vertebral dissemination within 6–8 months versus typically localized osteolytic lesions (10).

### 2.3 Diagnosis and endocrine therapy

Histopathological evaluation of the third biopsy specimen from the T10 vertebral lesion demonstrated poorly differentiated adenocarcinoma cells exhibiting cytokeratin positivity. Immunohistochemical profiling robustly supported a mammary origin, characterized by strong nuclear expression of estrogen receptor with 3+ intensity and 70% positivity, progesterone receptor with 3+ intensity and 70% positivity, HER-2 negativity at 1+ intensity, and a low Ki-67 proliferation index of 5%. While the low Ki-67 value suggested limited proliferative activity, potential sampling bias due to minimal tissue availability was acknowledged as a confounding factor. Further immunophenotypic analysis revealed CK5/6 negativity, excluding adenoepithelial differentiation, whereas CK7, mammaglobin, and GATA-3 positivity reinforced breast origin. Critical exclusion of alternative primary sites was achieved through PAX-8 negativity to rule out urothelial and renal carcinomas, CK20 and villin negativity to exclude gastrointestinal, pancreatic, biliary, and ovarian mucinous tumors, thyroglobulin and TTF-1 negativity to discard thyroid carcinoma, and vimentin negativity to eliminate mesenchymal neoplasms.

Synthesizing imaging, histopathology, and biomarker data, the metastatic lesions were conclusively attributed to advanced Luminal A breast cancer, defined by ER positivity, PR positivity, HER2 negativity, disseminated skeletal involvement, and absent visceral metastases. Given the incurable nature of widespread bone metastases and the endocrine sensitivity intrinsic to Luminal A subtypes, therapy was aligned with National Comprehensive Cancer Network (NCCN) guidelines. The regimen comprised fulvestrant, an estrogen receptor antagonist, combined with a CDK4/6 inhibitor such as palbociclib to target hormonal pathways and suppress cell cycle progression. Additionally, denosumab was administered to mitigate osteoclast-mediated bone destruction and reduce fracture risk, addressing the patient’s pathologic vertebral fracture.

### 2.4 Novel mutation in CHD4

Plasma NGS (580 genes) revealed a novel somatic CHD4 truncating mutation (c.2208G>A, p.Trp736Ter; VAF=4%), confirmed as pathogenic and previously undocumented. No pathogenic alterations were detected in key breast cancer drivers (PIK3CA/BRCA1/BRCA2/ERBB2/TP53), structural rearrangements, or CNVs. The p.Trp736Ter mutation causes truncation of the residues encoded by exon 15 (708–771), thereby preventing nucleosome disassembly and NuRD complex formation. TCGA analysis identified similar pathogenic truncating mutations (**Table 1**): adjacent variants associated with uterine malignancies (p.W728*/p.W741*), and upstream hinge region truncating mutations (p.684*/p.685*, etc.) associated with colorectal cancer/bladder cancer/endometrial cancer and melanoma. Additionally, similar truncating mutations in the subfamily homologous proteins CHD3 and CHD5 were summarized.

**Table 1:** Similar mutations in CHD3/4/5 across malignancies.

Gene	Age	Gender	Mutation location	Cancer type
CHD4	Unknown	Female	E752*	Lung Adenocarcinoma
	65	Female	R741*	Uterine Carcinosarcoma
	86	Female	G724*	Cutaneous Melanoma
	55	Female	R714*	Uterine Endometrioid Carcinoma
	60	Female	R714*	Breast Invasive Ductal Carcinoma
	Unknown	Unknown	R714*	Ampullary Carcinoma
	55	Female	R701*	Endometrial Carcinoma
	79/80	Male	E698*/E685*	Bladder Urothelial Carcinoma
	77	Male	R684*	Rectal Adenocarcinoma
	64	Male	D720Efs*11	Hepatocellular Carcinoma
CHD3	78	Female	E744*	Uterine Endometrioid Carcinoma
	57	Male	L715Ffs*7	Renal Non-Clear Cell Carcinoma
	60	Male	Y706Lfs*13	Prostate Adenocarcinoma
	78	Female	E685*	Uterine Endometrioid Carcinoma
	49	Female	E685*	Colon Adenocarcinoma
	Unknown	Unknown	R684*	Lung Adenocarcinoma
CHD5	13	Male	W710*	T-Lymphoblastic Leukemia/Lymphoma
	73	Male	Q704*	Hepatocellular Carcinoma
	67	Male	Q704*	Stomach Adenocarcinoma
	Unknown	Male	W691*	Melanoma
	62	Female	K670Sfs*120	Breast Invasive Lobular Carcinoma
	78	Female	E646*	Stomach Adenocarcinoma
	68	Female	E646*	Lung Adenocarcinoma

Information retrieved from www.cbioportal.org.

Mutations in the gene marked with “*” result in premature termination of transcription.

The CHD4 protein contains sequentially arranged domains: CHDNT, two PHDs, two chromodomains, SNF2 ATPase, Helicase domain, two DUFs, and CHDCT (11). Its N-terminal region (CHD4-N) is basic and binds both DNA and poly(ADP-ribose) (PAR), with significantly higher affinity for PAR. This specific PAR binding is essential for recruiting CHD4 to DNA damage site (12). Additionally, CHD4-N acts as a transcriptional activator, interacting directly with the BRG1 ATPase domain to promote gene expression and autoregulating its function through competitive binding to BRG1 (4).

## 3 Discussion

The oncogenic role of CHD4 mutations is validated across cancers—suppressing WNT antagonists through DNMT cooperation in colorectal cancer, governing repair-associated methylation dynamics in ovarian cancer, and enabling TERT promoter epigenetic remodeling in thyroid cancer—demonstrating broad tumorigenic influence through direct DNMT modulation and NuRD-dependent methylation/deacetylation (13–15). CHD4-NuRD drives breast cancer metastasis initiation by orchestrating EMT through MTA1/3-mediated E-cadherin suppression and β-catenin mislocalization, while antagonizing GATA3-dependent MET via chromatin occlusion, enabling early dissemination from small primaries (16–18). Beyond EMT, CHD4 amplifies metastasis through Wnt/β-catenin co-activation, HIF-enhanced hypoxic responses, RhoA/ROCK-driven migration, and immune evasion via EZH2-coupled H3K27me3 silencing of chemokines, while concurrently promoting chemoresistance via MEK/ERK overactivation—establishing CHD4 as a multi-mechanistic therapeutic target across cancers (14, 19, 20).

Despite unclear primary origin, widespread bone metastases occurred, warranting investigation into links with breast cancer molecular subtypes (especially high-risk Luminal tumors at 29.9%) and potential novel CHD4 mutations (21). Bone tropism is driven by specific genetic programs (e.g., RUNX2, IL11/CTGF/CXCR4) and the “seed and soil” hypothesis involving RANKL/TGF-β cascades (22). CHD4 drives breast cancer epigenetic dysregulation by recruiting HDAC1/2 and DNMTs via the NuRD complex to silence tumor suppressor genes, with its global impact manifested in breast cancer CpG island methylator phenotype (B-CIMP) linked to metastatic transcriptomes, ERα/immune dual-cluster regulatory networks, and replication-linked methylation clocks (23–25).

The role of CHD4 in gene silencing provides a key framework for explaining the carcinogenic effect of its mutations. Although the classic CHD4 mutation is believed to lead to loss of function, especially impounding its NURD-dependent gene silencing ability, thereby disrupting the inhibition of tumor suppressor genes, the research results may suggest a unique mechanism: The novel SNF2 domain truncation mutation (p.RP736TER) may lead to gene activation rather than simply eliminating the activity of CHD4. The molecular model of the mutant CHD4 confirmed that its N-terminal region retains a specific binding to the bromine domain of BRG1, and that this interaction mediates the gene activation function of CHD4 (4). This combination has the potential to disrupt the normal interaction between BRG1 and chromatin, thereby tilting CHD4 towards excessive transcriptional activation and simultaneously damaging its ATPase-dependent gene silencing function (due to SNF2 domain truncation). This functional imbalance, characterized by enhanced activation in conjunction with reduced silencing, has the potential to drive abnormal expression of oncogenes or metastasis-related genes. This, in turn, can promote the occurrence and progression of occult breast cancer with bone metastases. Consequently, it is imperative to ascertain the epigenetic consequences of this mutation and its impact on BRG1 binding.

## Data Availability

The original contributions presented in the study are included in the article/**Supplementary Material**. Further inquiries can be directed to the corresponding author.
