# Supplementary figures and images for: Case Report: Novel mutation in CHD4 triggers occult breast cancer with bone metastases
Source: Front Oncol. 2025 Sep 29;15:1682794. doi: 10.3389/fonc.2025.1682794 (PMC12515663; doi:10.3389/fonc.2025.1682794)

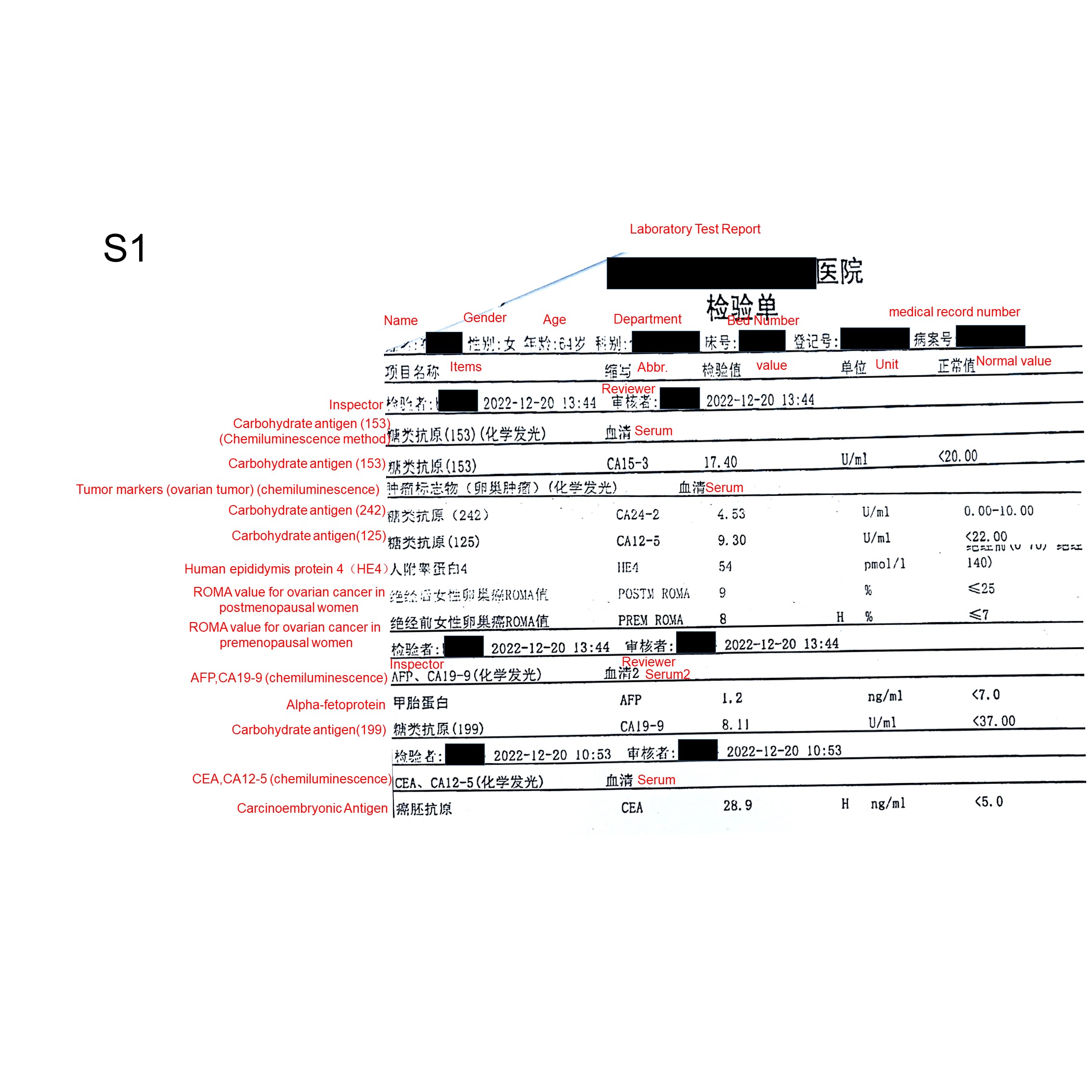

Supplement: Supplementary file 1 [file Image1.jpg]

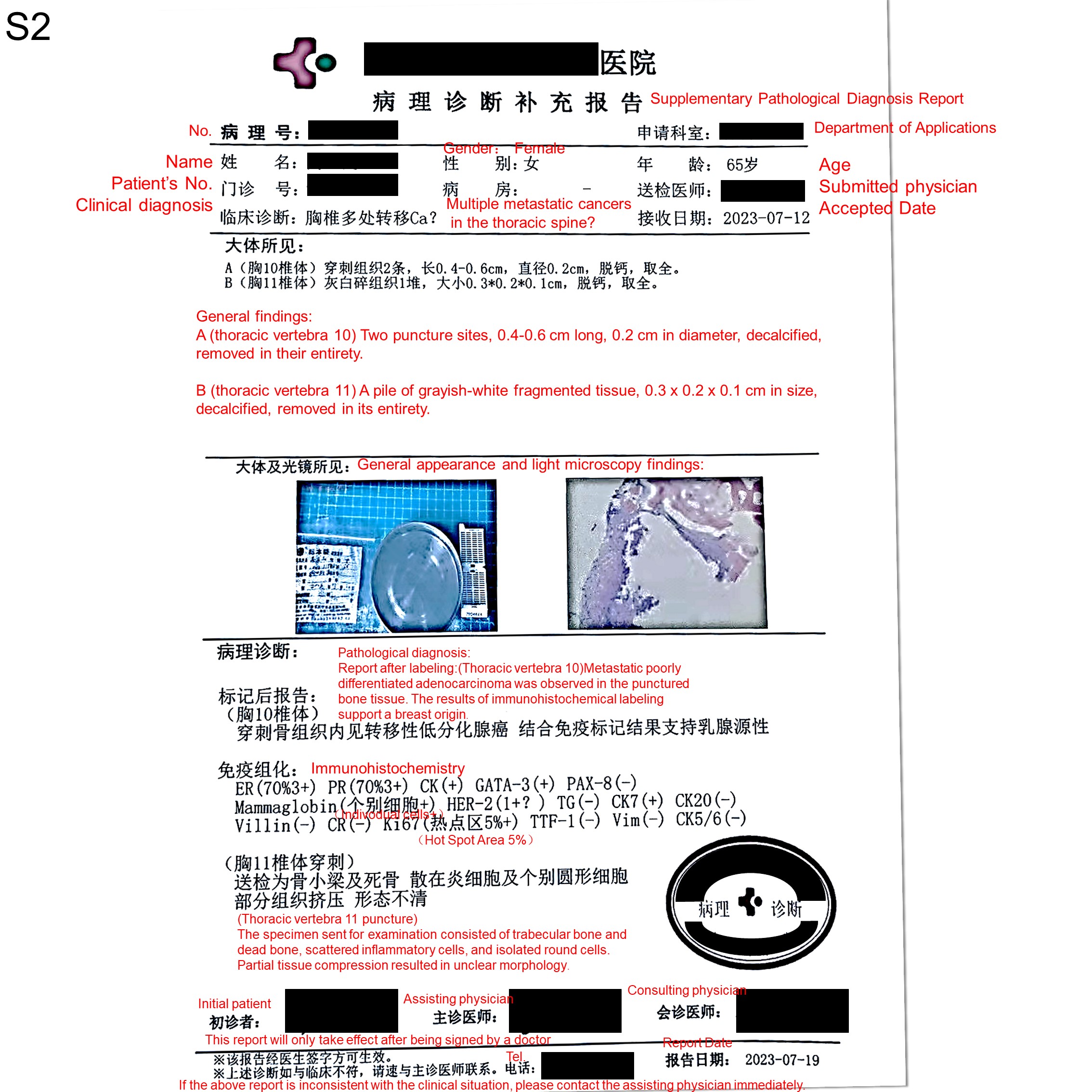

Supplement: Supplementary file 2 [file Image2.jpg]

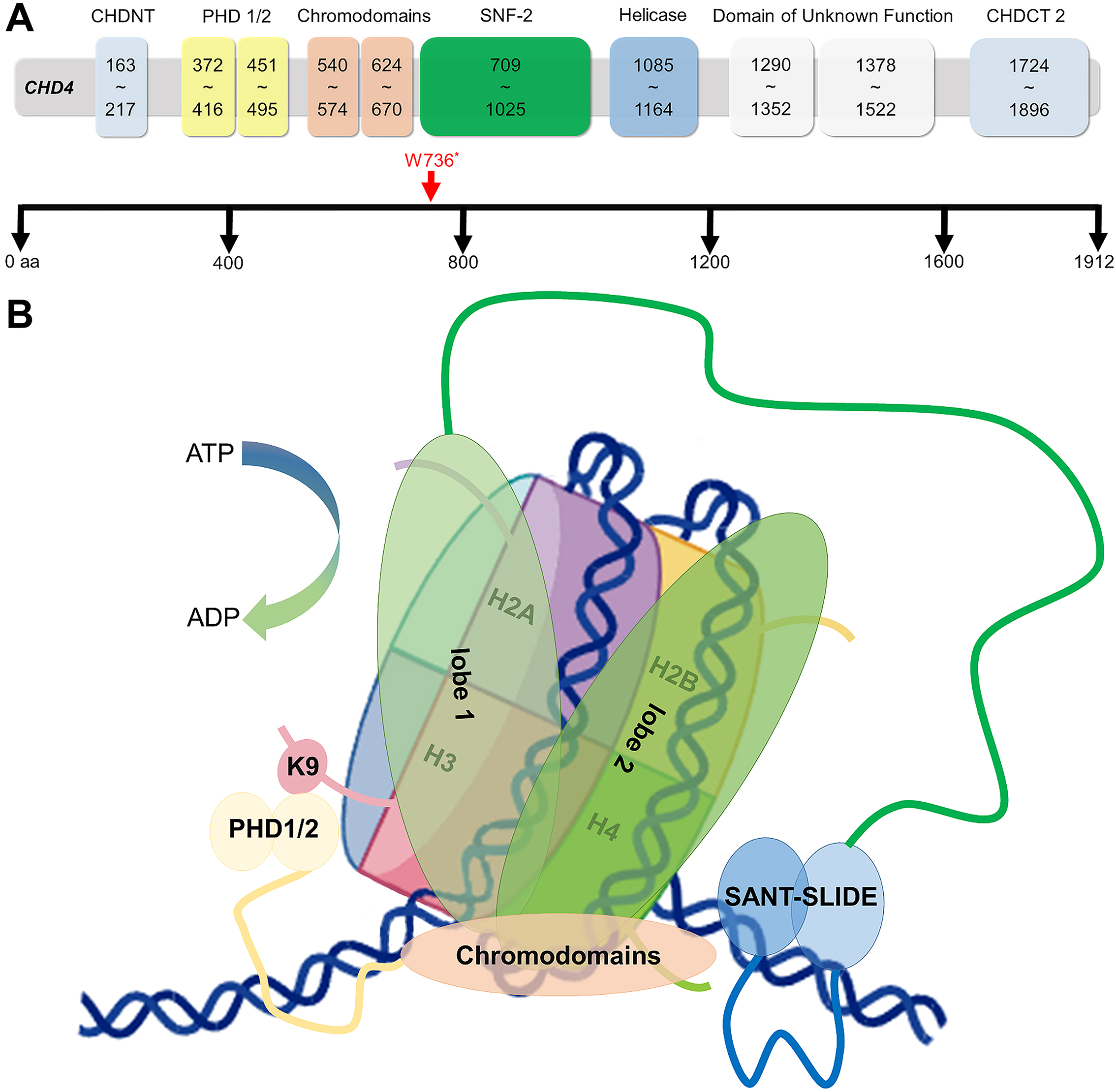

Supplement: Supplementary file 3 [file Image3.jpeg]

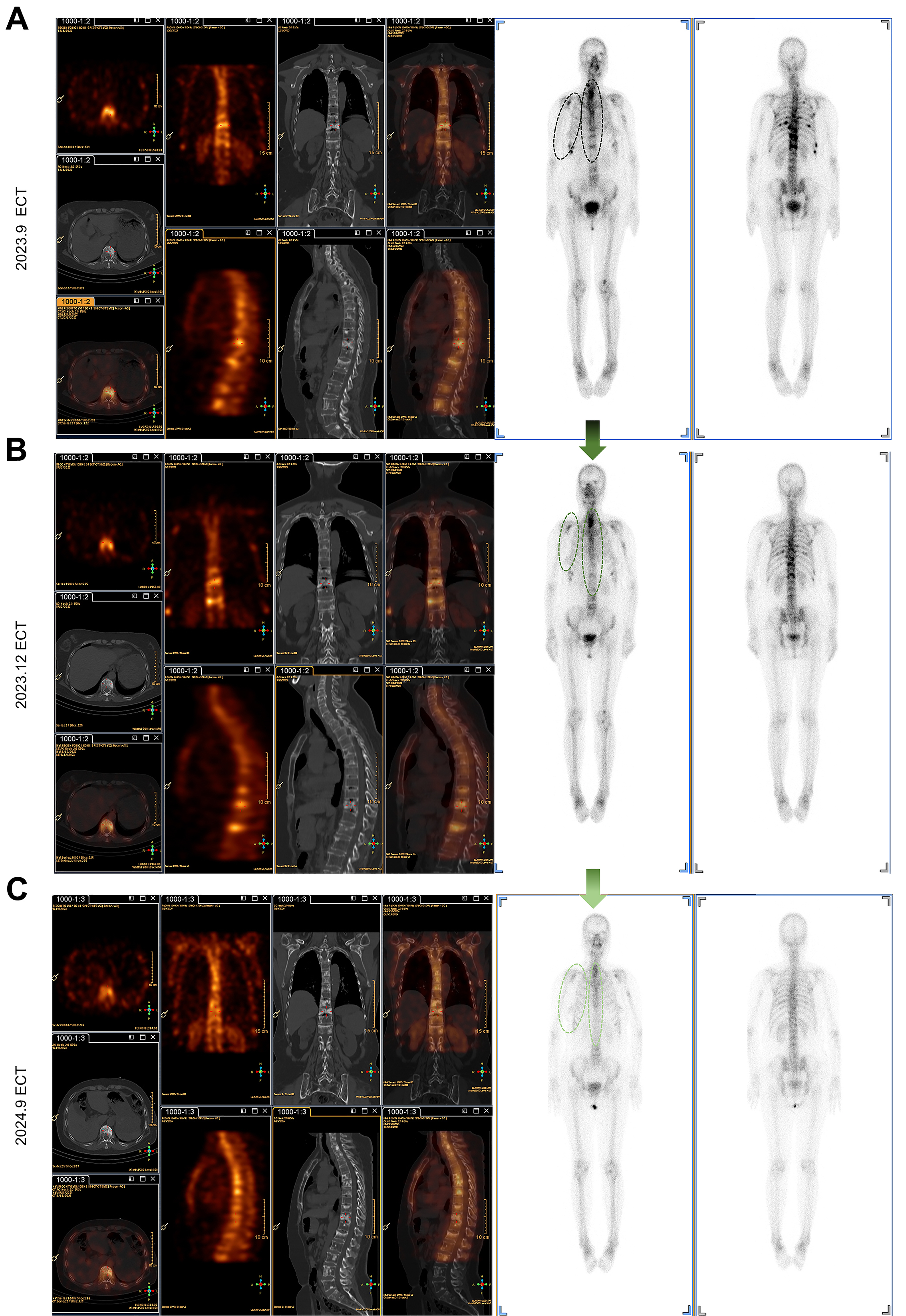

Supplement: Supplementary file 4 [file Image4.jpeg]

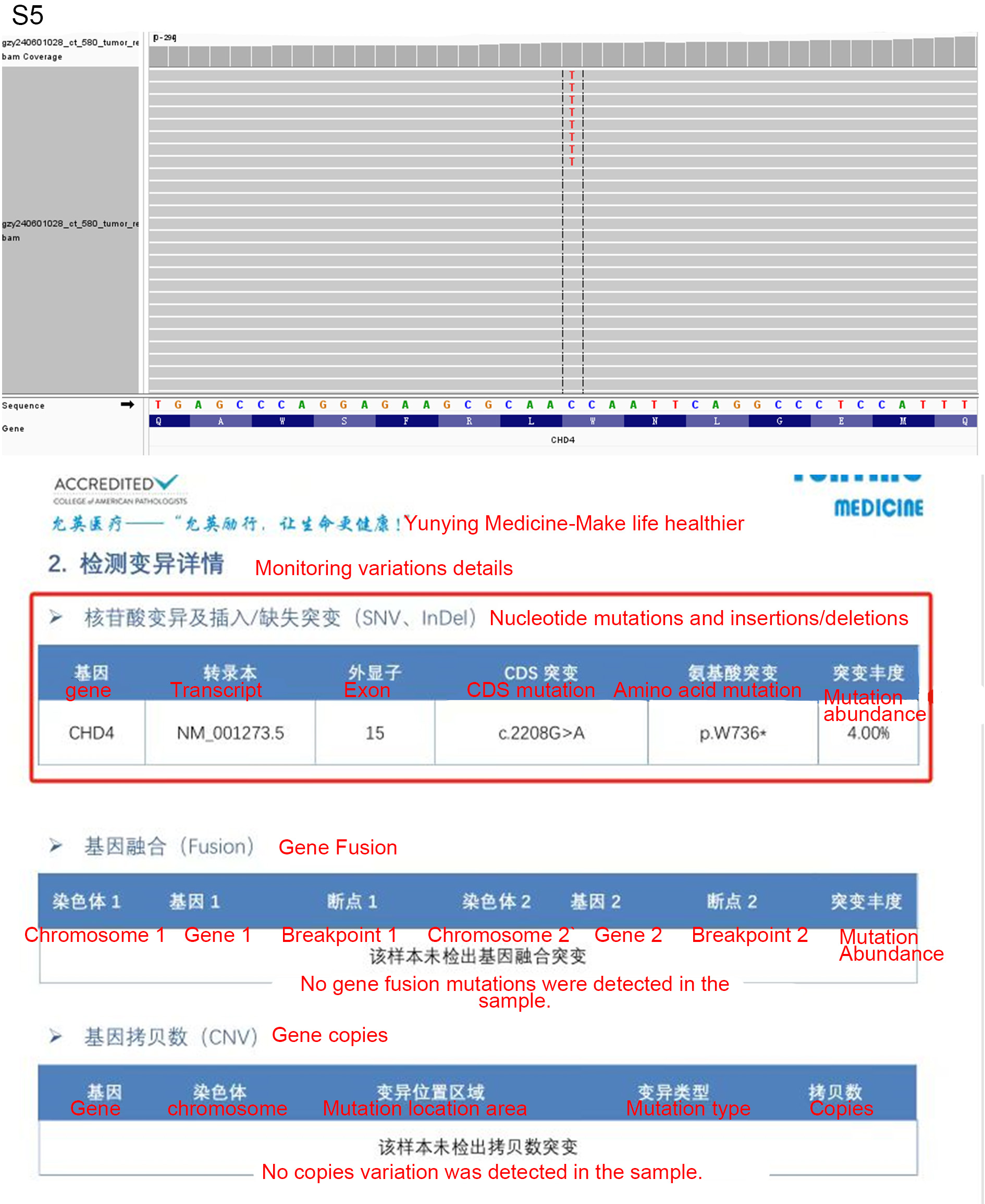

Supplement: Supplementary file 5 [file Image5.jpg]
